# New Media Use by Patients Who Are Homeless: The Potential of mHealth to Build Connectivity

**DOI:** 10.2196/jmir.2724

**Published:** 2013-09-03

**Authors:** Lori Ann Post, Federico E Vaca, Kelly M Doran, Cali Luco, Matthew Naftilan, James Dziura, Cynthia Brandt, Steven Bernstein, Liudvikas Jagminas, Gail D'Onofrio

**Affiliations:** ^1^Department of Emergency MedicineYale School of MedicineYale UniversityNew Haven, CTUnited States; ^2^Department of Emergency Medicine and Department of Population HealthNYU School of MedicineNew York UniversityNew York, NYUnited States

**Keywords:** homelessness, mHealth, connectivity, emergency department

## Abstract

**Background:**

Patients experiencing homelessness represent a disproportionate share of emergency department (ED) visits due to poor access to primary care and high levels of unmet health care needs. This is in part due to the difficulty of communicating and following up with patients who are experiencing homelessness.

**Objective:**

To determine the prevalence and types of “new media” use among ED patients who experience homelessness.

**Methods:**

This was a cross-sectional observational study with sequential enrolling of patients from three emergency departments 24/7 for 6 weeks. In total, 5788 ED patients were enrolled, of whom 249 experienced homelessness. Analyses included descriptive statistics, and unadjusted and adjusted odds ratios.

**Results:**

70.7% (176/249) of patients experiencing homelessness own cell phones compared to 85.90% (4758/5539) of patients in stable housing (*P*=.001) with the former more likely to own Androids, 70% (53/76) versus 43.89% (1064/2424), and the latter more likely to have iPhones, 44.55% (1080/2424) versus 17% (13/76) (*P*=.001). There is no significant difference in new media use, modality, or frequency for both groups; however, there is a difference in contract plan with 50.02% (2380/4758) of stably housed patients having unlimited minutes versus 37.5% (66/176) of homeless patients. 19.78% (941/4758) of patients in stable housing have pay-as-you-go plans versus 33.0% (58/176) of homeless patients (*P*=.001). Patients experiencing homelessness are more likely to want health information on alcohol/substance abuse, mental health, domestic violence, pregnancy and smoking cessation.

**Conclusions:**

This study is unique in its characterization of new media ownership and use among ED patients experiencing homelessness. New media is a powerful tool to connect patients experiencing homelessness to health care.

## Introduction

### Background

Patients who are homeless experience high levels of unmet health needs [1] and poor access to primary care [2]. Thus it is not surprising that people who are homeless represent a disproportionate share of emergency department (ED) patients [3]. Beyond accessing the ED for health care, they are also motivated by social needs such as food, shelter, and safety [4]. Communication and follow-up with ED patients experiencing homelessness is a major barrier. Such connectivity needs led us to explore “new media” as a means to better serve patients experiencing homelessness who routinely access the ED for their health care.

### What Is New Media?

New media refers to on-demand access to content anytime, anywhere, using a digital device that includes interactive user feedback, creative participation, and community formation around the media content [5] and has characteristics of being manipulated, networkable, dense, compressible, and most importantly, interactive [6]. Examples of new media include the Internet, social networking websites, multimedia, video games, cell phones, and smart phones [6], as opposed to legacy media such as television, radio, film, magazines, or paper-based publications, unless they contain technologies that enable digital interactivity [7,8]. For the purpose of this study, mHealth is defined as “the delivery of health care services via mobile communication devices” [9].

Connectivity, identified by mHealth researchers, is crucial between patients, providers, and the system of care [10,11] prompting the Federal Communication Commission to create a task force on mHealth. The overarching goal given to the task force was to identify necessary steps to attain the following: “By 2017 mHealth, wireless health and e-Care solutions will be routinely available as part of best practices for medical care” [12]. What is missing from US health care goals is how to include patients who are experiencing homelessness in these important plans. To this end, this study attempts to determine whether the use of new media can improve the ED health care of patients experiencing homelessness and transcend health care service delivery barriers through connectivity.

### What Are the Challenges in the Emergency Department of Treating Patients Experiencing Homelessness?

We must address issues of homelessness because they constitute a particularly vulnerable population of patients [3]. Patients without access to primary care have few alternatives, which contributes to overcrowding and nonemergency care being provided in the ED [4,13-18]. ED practitioners find that social needs must often be addressed before they can begin to address these patients’ health care.

### Connectivity for Patients Experiencing Homelessness?

The realization that new media might serve a powerful function in the care and well-being of patients who are homeless has been described more recently in a handful of studies [19-24], nor is it missing from the enhancement of health care outside the context of homelessness [25-28]. In fact, increasing connectivity through new media has been practiced globally for nearly two decades [29-33]. However, what is not known is the use of new media by patients in the ED who are experiencing homelessness and how this compares to other ED patients or the general population. Prior studies of adults who were homeless found that 44%-54% had cellular phones, but these studies were limited by using geographically limited convenience samples and were not specific to ED patients [24,34]. Ranney et al’s study was the first to describe overall ED patients’ preferences for technology-based interventions and the first to develop baseline data on use of computers, Internet, cell phone, and SMS text messaging, but they did not examine this in patients who were homeless [35]. The current study goes beyond their work by identifying patients who were homeless and differentiating the various modalities of new media beyond cell phone use derived from the communication literature [35].

## Methods

### Design

This study was an observational cross-sectional survey that continuously enrolled sequential patients in three EDs 24 hours per day, 7 days per week for 6 weeks (July-August 2012).

### Setting

Patients were enrolled from three urban, high-volume EDs (Connecticut, USA) at Yale-New Haven Hospital (n=1922), Bridgeport Hospital (n=1900), and Hospital of St. Raphael (n=1966) for a total of 5788 patients.

### Participants

**Figure 1:**
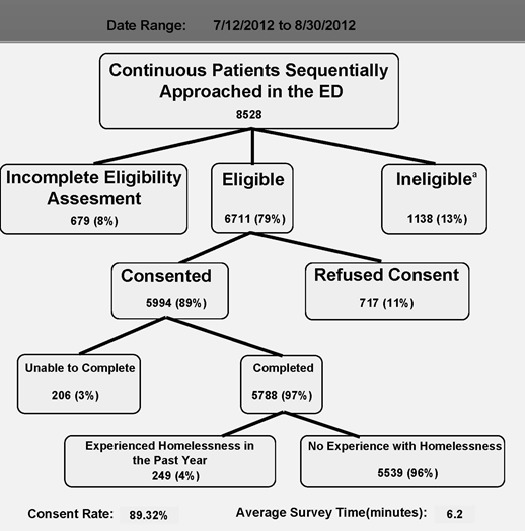
Patient flow diagram.

Patients were excluded if they were under 18 years of age; presented as a trauma activation; presented with alcohol or other substance intoxication; spoke a language other than English or Spanish; presented with active psychosis, suicidal, or homicidal ideation; were in police custody, unable to consent due to life-threatening events or cognitive impairment; were in isolation for infectious concerns until cleared by provider; or were unable/unwilling to consent (Figure 1). For some prospective participants, study enrollment was delayed due to initial limited decisional capacity secondary to alcohol and or other substances, but they were approached at a later time. Overall, 89% of eligible patients consented to participate in this study (Figure 1). Patients were interviewed by trained research assistants.

### Data Analyses

Our analysis included descriptive statistics, bivariate data analysis, and unadjusted and adjusted odds ratios.

### Measures

Patients were asked a series of questions on homelessness: (1) how many nights they spent in their own home during the last week, (2) how many nights they spent at somebody else’s house, in a motel, in a half-way house, in transitional housing, in an institution, in jail, in shelter, and outdoors, and (3) where else they stayed in the past week (to rule out vacations or family and friend visits that were recreational vs shelter seeking). After reviewing the different potential options, we asked also patients to (4) estimate the number of times they had been homeless in the past year. We used a broad definition of homelessness, which included patients living “doubled up” with family or friends, or in some other transitional living arrangement such as staying in a motel, at their place of work, in a church, or a car in addition to including patients who were living in shelters or on the streets or other public places not meant for nighttime residence. This definition is consistent with that used by the US Health Resources and Services Administration (HRSA) in providing guidance to Health Care for the Homeless centers [36] and was chosen to be inclusive of the broad spectrum of people vulnerable to the health risks associated with homelessness. In addition, because homelessness is most commonly a transient state, with people cycling into and out of homelessness or experiencing short episodes of homelessness [37], we included as homeless any patient who indicated an episode of homelessness over the past year.

Patients were also asked (5) if they owned a cell phone, (6) what their cell phone was used for, including phone calls, text messaging, emailing, surfing the Internet, watching videos, listening to music, playing games, applications, and other, (7) what type of phone, provider, phone plan they had, and (8) frequency of use. Patients were asked about new media behaviors such as (9) seeking health information, and (10) tracking and managing health through a personal health record (PHR) or other application. Patients were asked about (11) use of computers, access, where accessed, and ownership. They were also asked about (12) accessing the Internet through cell phone, laptop, desktop, tablet; frequency of use; duration of use per day; purposes of use; social networking; and chatting; and (13) if would they be interested in receiving health information via each type of media about a variety of health issues. We included open-ended questions where patients could suggest other health topics of interest outside of those listed in the survey. The study ended with (14) the collection of demographics, health issues, access to health care, and insurance status.

## Results

In total, 5788 subjects were enrolled in the study. Of these, 249 (4.30%) patients reported episodes of homelessness in the past year. Patients who had experienced homelessness were more likely to be male (54.6%, 136/249), younger (mean age 40 vs 46 years), African American (38.6%, 96/249) or Latino (25.3%, 63/249), and have lower income and less education than stably housed patients (Table 1).

Patients with a history of homelessness reported similar types of new media use as stably housed patients in terms of making phone calls, text messaging, emailing, surfing the Internet, social networking, using PHRs, and looking up health information (Table 2). Fewer homeless patients owned cell phones (70.7%, 176/249 vs 85.90%, 4758/5539; *P*=.001) or smart phones (43.2%, 76/176 vs 50.95%, 2424/4758; *P*=.04) as compared to the non-homeless ED patients (Table 2). Patients experiencing homelessness did own significantly different types of smartphones (*P*=.001) and had different types of cell phone and smart phone contracts (*P*=.001) compared to stably housed patients. Stably housed patients were more likely to own iPhones (44.55%, 1080/2424; *P=*.001) and have a contract plan with unlimited minutes (50.02%, 2380/4758; *P*=.001) whereas, patients who were homeless were more likely to own Android phones (70%, 53/76; *P*=.001) and own “pay-as-you-go” plans (33.0%, 58/176; *P*=.001).

Among those who owned a cell or smartphone, patients experiencing homelessness were slightly more likely to look up health information (64%, 52/81 vs 59.81%, 1317/2202) or track and manage their health using a PHR (20%, 16/81 vs 18.26%, 402/2202); however, these differences were not statistically significant (Table 2).

Regardless of media use, we questioned patients about their need and desire for health information. Table 3 shows unadjusted odds ratios (OR) comparing the desire for health information in patients experiencing homelessness to housing-stable patients. Patients experiencing homelessness were significantly more likely to want health information on mental health (OR 2.2), smoking cessation (OR 3.0), alcohol abuse (OR 1.9), pregnancy (OR 1.4), drugs/substance abuse (OR 2.8), and domestic violence (OR 2.4). When evaluating only smokers using adjusted odds ratios, homeless patients were still significantly more likely to want health information about smoking cessation than non-homeless patients (*P*=.01). Patients experiencing homelessness were similar to stably housed patients in their desire for health information on weight loss/nutrition and managing chronic diseases such as hypertension or diabetes. We also asked patients which other topics they would be interested in receiving health information about. Patients experiencing homelessness listed dozens of additional topics such as HIV, diabetes, heart disease, epilepsy, pain, fall prevention, cancer, health insurance, health care options, kidney stones, menopause, and multiple sclerosis to name a few.

**Table 1:** Demographics—Emergency Department Media Study, July 12-August 30, 2012.

Characteristics		Participants who were homeless ≥1 times in last year (n=249), n (%)^a^	Participants who were not homeless in last year (n=5539), n (%)^b^
**Gender**			
	Men	136 (54.6)	2270 (40.98)
	Women	113 (45.4)	3269 (59.02)
**Age**			
	18-29	78 (31.3)	1431 (25.83)
	30-49	100 (40.2)	1939 (35.01)
	50-64	59 (23.6)	1106 (19.97)
	65+	12 (4.8)	1063 (19.19)
**Race/ethnicity**			
	White, non-Hispanic	90 (36.1)	2320 (41.88)
	Black, non-Hispanic	96 (38.6)	1855 (33.49)
	Hispanic	63 (25.3)	1295 (23.38)
**Annual household income**			
	Less than $30,000/yr	193 (91.9)	2691 (62.64)
	$30,000-$59,999	10 (4.8)	818 (19.04)
	$60,000-$89,999	4 (1.9)	436 (10.15)
	$90,000+	3 (1.4)	352 (8.19)
**Education level**			
	No high school diploma	93 (37.3)	752 (13.58)
	High school grad	97 (39.0)	2353 (42.48)
	Some college	39 (15.7)	1350 (24.37)
	College+	20 (8.0)	1084 (19.57)

^a^Participants who reported being homeless one or more times in the last year.

^b^Participants who reported not being homeless at any time in the last year.

**Table 2:** Media usage by ED patients experiencing homelessness.

			Homeless ≥1 times in last year (n=249), n (%)	Not homeless in last year (n=5539), n (%)	*P* value
**Cell phone ownership (% of total)**			176 (70.7)	4758 (85.90)	<.001
	Making phone calls (% of cell users)		175 (99.4)	4717 (99.14)	1
	Text messaging (% of cell users)		126 (71.6)	3469 (72.91)	.7
	**Surfing the Internet (% of cell users)**		81 (46.0)	2202 (46.27)	.95
		Look up health information (% of cell phone surfers)	52 (64.2)	1317 (59.81)	.43
		Track or manage health with app (% of cell phone surfers)	16 (19.8)	402 (18.26)	.73
	Emailing (% of cell users)		74 (42.0)	2007 (42.18)	.97
	Social networking (% of cell users)		67 (38.1)	1836 (38.59)	.89
	Listening to music (% of cell users)		65 (36.9)	1503 (31.59)	.14
	Playing games (% of cell users)		61 (34.7)	1355 (28.48)	.08
	Using apps (% of cell users)		57 (32.4)	1436 (30.18)	.53
	Watching online videos (% of cell users)		48 (27.3)	1294 (27.20)	.98
	Smartphone ownership (% of cell users)		76 (43.2)	2424 (50.95)	.043
	**Type of smartphone (% of smartphone owners)**				<.001
		Android	53 (69.7)	1064 (43.89)	
		iPhone	13 (17.1)	1080 (44.55)	
		Blackberry	7 (9.2)	171 (7.05)	
		Windows	3 (3.9)	52 (2.15)	
		Other	0 (0.0)	57 (2.35)	
**Type of cell phone plan (% of cell users)**					<.001
	Contract plan with unlimited minutes		66 (37.5)	2380 (50.02)	
	Pay-as-you-go plan		58 (33.0)	941 (19.78)	
	Contract plan with limited minutes		35 (19.9)	1239 (26.04)	
	Medicaid phone		16 (9.1)	197 (4.14)	
	Other		1 (<1.0)	1 (<1.00)	
**Internet use (% of total)**			147 (59.0)	3767 (68.00)	.003
	Email use (% of Internet users)		113 (76.9)	3173 (84.23)	.017
	Social networking use (% of Internet users)		102 (69.4)	2606 (69.18)	.96

**Table 3:** Desire for health information by ED patients experiencing homelessness (% of adults in each group with desire for various health information).

“If we offered you free health information, which topics would you be interested in receiving?”(Check all that apply)	Homeless ≥1 times^a^, n (% of total n=249 who said “Yes”)	Homeless 0 times^a^, n (% of total n=5531 who said “Yes”)	OR	95% CI
Healthy weight / nutrition / weight loss	156 (62.7)	3626 (65.56)	0.88	0.67-1.14
Mental health	125 (50.2)	1755 (31.73)	2.16	1.68-2.79
Smoking	106 (42.6)	1101 (19.91)	2.98	2.30-3.86
Alcohol	54 (21.7)	719 (13.00)	1.85	1.35-2.53
Pregnancy	39 (15.7)	639 (11.55)	1.42	1.00-2.02
Drugs / substance abuse	59 (23.7)	553 (10.00)	2.79	2.06-3.79
Domestic violence	47 (18.9)	490 (8.86)	2.39	1.72-3.33
Managing chronic disease	119 (47.8)	2518 (45.53)	1.09	0.84-1.41

^a^Participants were asked: “How many times have you been homeless in the last year?”

## Discussion

### Principal Findings

This study provides the first estimates of new media use among ED patients experiencing homelessness. Surprisingly, overall new media ownership by ED patients is similar to that in the general population and only slightly higher than the media ownership by ED patients experiencing homelessness [38]. As 70.7% (176/249) of the homeless ED population already own cell phones with the ability to text and receive calls, ED providers can increase connectivity for all mHealth purposes including referrals, appointment and medication reminders, and providing relevant information for health management. We also found that the type of smartphone and cell phone contracts or plans were significantly different for these two ED populations, which has implications for health care and research. Given that patients who are homeless are more likely to pay-as-you-go rather than enter into a long-term contract, health care providers need to be cognizant of limitations. One solution to these limitations would be to provide patients experiencing homelessness with “minutes” to increase their connectivity for health care service and research purposes. The rates of cell phone ownership from this study are higher than those observed in prior convenience samples of adults who were homeless, which found that 44%-54% had cellular phones [24,34]. This difference may be due to sampling and definition of homelessness. Our study included a broad definition of homelessness including patients who were transiently homeless in addition to the chronically homeless. Regardless, the high rates of new media ownership, access, and use observed among ED patients experiencing homelessness suggest that providers can use this technology to communicate with patients who are homeless [38], which was unknown until this study.

Importantly, patients experiencing homelessness were similar to stably housed patients in types of new media use, modes of media, and frequency of use, defying popular assumptions of a large “digital divide” for patients who are homeless. This finding is consistent with prior research showing that young adults who were homeless versus non-homeless had very similar uses of social network technology [39] and suggests that such similarities may extend to older adults as well. In addition, homeless patients are similar to stably housed patients in “new media use”, meaning they should not be thought of as different or unique from other patients. Both populations use mobile devices, both know their functionality, and both can benefit from mHealth. Thus, patients without a home are not remarkably different from those who were more stably housed in terms of media use and media knowledge. However, it is true that they are more likely to have problems with substance abuse and mental health with limited access to health care [40]. Patients experiencing homelessness often feel that they are treated as inferior when interacting with the health care system [41]. Interestingly, new media may be a strong facilitator of health equity. Those identifying as homeless were slightly more likely to look up health information (64%, 52/81) than stably housed ED patients (59.81%, 1317/2202) and twice as likely to look up health information than the general population as reported in previous studies (31%) [38]. While health status may be driving the health information-seeking behavior, we still find evidence that homeless patients are higher new media users and information seekers even when controlling for baseline health status factors; for example, even when controlling for smoking status, patients who were homeless were still more likely to desire information on smoking cessation. Thus, as new media use is a powerful tool in health care for all patient populations, this connectivity may be an even more important tool for homeless patients because they may not have access to health information from primary care providers or be exposed to health information through formal education. Smartphones can meet their Internet and application needs as they relate to health care when stable housing with landlines, desktops, laptops, and Wi-Fi access are not available. Cellular phones and smartphones are portable, offering connectivity regardless of where the patient resides or how often they move. Finally, new media offers health care providers an opportunity to connect with their homeless patients, leveraging mobile technology to improve patient health outcomes. The findings of this study suggest programs such as Lifeline or LinkUp America—Medicaid programs that provide cell phones/land lines for impoverished patients without health insurance—do indeed increase connectivity with health care providers [42].

Finally, ED patients experiencing homelessness were significantly more likely to want information on chronic health and social problems such as mental health, smoking cessation, alcohol and other substance abuse, pregnancy, and domestic violence than their stably housed counterparts. Negative consequences of these conditions are often treated in the ED, and preventative interventions may in fact decrease ED visits, health care costs, and improve health.

### Limitations

One limitation of our study is that patients who were intoxicated for long periods of time and/or actively psychotic were unable to give informed consent. This may have resulted in certain subsegments of ED patients who are homeless to be excluded from the study. In particular, patients who are chronically homeless suffer from disproportionately high levels of substance abuse and mental health disorders and thus may be underrepresented in the current study.

### Conclusion

In summary, ED patients experiencing homelessness have high rates of cell phone ownership and are equal in new media use to stably housed patients adjusting for ownership. They are more likely to engage in all forms of mHealth. Our expanded knowledge about the desire for connectivity by patients who are homeless informs opportunities for prevention and intervention to improve the health of this vulnerable population and potentially decrease the cost of health care.

## Abbreviations

ED: emergency department
HRSA: Health Resources and Services Administration
PHR: personal health record
